# Incubation of Horseradish Peroxidase near 50 Hz AC Equipment Promotes Its Disaggregation and Enzymatic Activity

**DOI:** 10.3390/mi16030344

**Published:** 2025-03-19

**Authors:** Yuri D. Ivanov, Ivan D. Shumov, Andrey F. Kozlov, Alexander N. Ableev, Angelina V. Vinogradova, Ekaterina D. Nevedrova, Oleg N. Afonin, Dmitry D. Zhdanov, Vadim Y. Tatur, Andrei A. Lukyanitsa, Nina D. Ivanova, Evgeniy S. Yushkov, Dmitry V. Enikeev, Vladimir A. Konev, Vadim S. Ziborov

**Affiliations:** 1 Institute of Biomedical Chemistry, Pogodinskaya Str., 10 Build. 8, 119121 Moscow, Russia; shum230988@mail.ru (I.D.S.); afkozlow@mail.ru (A.F.K.); ableev@mail.ru (A.N.A.); angeluna1234@bk.ru (A.V.V.); nevedrova.kat@yandex.ru (E.D.N.); sunweb@mail.ru (O.N.A.); zhdanovdd@gmail.com (D.D.Z.); ziborov.vs@yandex.ru (V.S.Z.); 2 Joint Institute for High Temperatures of the Russian Academy of Sciences, 125412 Moscow, Russia; 3 Foundation of Perspective Technologies and Novations, 115682 Moscow, Russia; v_tatur@mail.ru (V.Y.T.); andrei_luk@mail.ru (A.A.L.); ninaivan1972@gmail.com (N.D.I.); 4 Faculty of Computational Mathematics and Cybernetics, Moscow State University, 119991 Moscow, Russia; 5 Moscow State Academy of Veterinary Medicine and Biotechnology Named after Skryabin, 109472 Moscow, Russia; 6 Department for Business Project Management, National Research Nuclear University “MEPhI”, 115409 Moscow, Russia; esyushkov@mephi.ru; 7 Institute for Urology and Reproductive Health, I.M. Sechenov First Moscow State Medical University (Sechenov University), 119991 Moscow, Russia; dvenikeev@gmail.com; 8 Department of Infectious Diseases in Children, Faculty of Pediatrics, N.I. Pirogov Russian National Research Medical University, 117997 Moscow, Russia; konev60@mail.ru

**Keywords:** low-frequency electromagnetic field, horseradish peroxidase, AC transformer, atomic force microscopy, enzymatic activity, enzyme disaggregation

## Abstract

Low-frequency electromagnetic fields, induced by alternating current (AC)-based equipment such as transformers, are known to influence the physicochemical properties and function of enzymes, including their catalytic activity. Herein, we have investigated how incubation near a 50 Hz AC autotransformer influences the physicochemical properties of horseradish peroxidase (HRP), by atomic force microscopy (AFM) and spectrophotometry. We found that a half-hour-long incubation of the enzyme above the coil of a loaded autotransformer promoted the adsorption of the monomeric form of HRP on mica, enhancing the number of adsorbed enzyme particles by two orders of magnitude in comparison with the control sample. Most interestingly, the incubation of HRP above the switched-off transformer, which was unplugged from the mains power supply, for the same period of time was also found to cause a disaggregation of the enzyme. Notably, an increase in the activity of HRP against ABTS was observed in both cases. We hope that the interesting effects reported will emphasize the importance of consideration of the influence of low-frequency electromagnetic fields on enzymes in the design of laboratory and industrial equipment intended for operation with enzyme systems. The effects revealed in our study indicate the importance of proper shielding of AC-based transformers in order to avoid the undesirable influence of low-frequency electromagnetic fields induced by these transformers on humans.

## 1. Introduction

Electricity has become a part and parcel of modern life, being ubiquitously employed both in industry and for household use. Currently, alternating current (AC)-based equipment is used most widely [1,2]. One main advantage of AC is the transformability of AC voltage [1]. This allows one to avoid heat loss by using high-voltage AC lines and circuits, thus making AC electric-power transmission preferable owing to its cost efficiency [1]. Accordingly, electric AC transformers represent key components of AC lines and equipment. In Europe, the commercial AC frequency is 50 Hz, pertaining to a low frequency range [1,2]. In Northern America, a 60 Hz commercial frequency is employed [1]. The operation of AC equipment, including AC transformers, is known to be accompanied by the induction of electromagnetic fields of respective frequency (low-frequency electromagnetic fields, LFFs). Low-frequency magnetic and electromagnetic fields are known to influence the physicochemical properties and functioning of enzymes [2,3,4,5,6,7]. Typically, the exposure of enzymes to AC equipment occurs in bioreactors with motor-driven stirring devices [8,9]. Of course, this is just the most illustrative case, since it is also common for LFFs to affect AC equipment operators, while the processes in the human body are known to be regulated by enzymes [10]. The impact of electromagnetic fields on the body and, in particular, on enzymes has been analyzed in many works [11,12,13,14,15,16,17]. Of course, the exact effect of an external field on an enzyme depends on the type of the enzyme and the parameters of the field [2,4,18], and the variety of important enzymes is quite wide [10]. The evident effects of external fields, including LFFs, on enzymes [2,3,4,5,6,7] thus motivate researchers to further study these phenomena.

In the literature, particular attention has been paid to the effects of magnetic and electromagnetic fields on the horseradish peroxidase (HRP) enzyme [2,3,4,5,6,7,11,18]. This enzyme has found numerous practical applications in biotechnology as a useful catalyst [19]. For instance, the uses of HRP for wastewater purification [20], in food technology [21] and in biofuel cells [22,23,24] have been reported. Furthermore, HRP is used in healthcare as a reporter enzyme in diagnostic systems [25,26]. This is why this enzyme attracts particular attention from scientists. The enzymatic activity of HRP was shown to change significantly under the action of electromagnetic fields [21], including LFFs [2,3,5]. Since LFFs are induced by various industrial AC-energized equipment (for instance, by transformers and electric motors) employed in biotechnological setups, a detailed investigation of their influence on the functionality of HRP is evidently required. Furthermore, the adsorption/aggregation properties of HRP were found to be quite sensitive to the influence of magnetic and electromagnetic fields [2,6,7,27,28,29]. Given the latter, this enzyme can be used as an electromagnetic radiation sensor [6,7,29]. To this end, the sensitivity of methods employed for the detection of changes in the enzyme’s properties has become a key point of study [27,28].

In studies of enzymes, various spectroscopy-based methods are commonly employed [11,18,30,31]. These methods are, however, only helpful when the enzyme under study contains a chromophoric group (for instance, in cases of cytochromes P450), or when changes in the enzyme’s spatial structure [11,18] and/or functional activity [2,3,18,28] are significant. A loss of activity often occurs due to denaturation [32]. Gajardo-Parra et al. [33] reported that the activity of HRP correlates with the α-helix content in its spatial structure. The denaturation of HRP can take place upon the action of chemical agents [32], pulsed light [34], high (70 °C and higher) temperatures [21,35] and microwave radiation [36]. Considering peroxidases in general, radio frequency [37] and microwave [38] radiation and various types of electric fields [39,40,41,42] were also reported to cause enzyme inactivation. This inactivation can also be ascribed to enzyme denaturation [40,41,42]. Indeed, stabilization of the spatial structure of HRP was shown to prevent its irreversible denaturation-caused inactivation [35]. The denaturation-caused inactivation of peroxidases can be unambiguously revealed by spectroscopy-based methods [34,35,37,39,40,41,42].

At the same time, the changes in the enzyme’s properties are often quite subtle, and, hence, are barely distinguishable [18] or completely indistinguishable [27] by spectroscopic methods. These changes can, nevertheless, be important with regard to enzyme functionality [28]. If this is the case, high-resolution methods are required in order to perform single-molecule investigations of the enzymes of interest. One well-known method for the high-resolution visualization of various objects of micron and sub-micron size is electron microscopy [43,44]. Transmission electron microscopy enables the visualization of studied specimens with sub-nanometer resolution, as was recently demonstrated for inorganic matter by Yang et al. [43]. Electron microscopy visualization of proteins, however, implies the use of harsh conditions (negative staining [45,46] or so-called vitreous ice [46]), which are far from native ones. In this respect, atomic force microscopy (AFM) is quite helpful [6,7,27,28,29]. Tapping-mode AFM enables the impact of AFM probes on the studied sample to be minimized upon the visualization of single enzyme molecules, providing their visualization under near-native conditions [47], thus allowing scientists to reveal even subtle changes in the enzyme properties [6,7,27,28]. The parallel use of AFM and spectroscopic methods is also a good practice [27,28,29].

In the work presented, the effect of incubation of the HRP solution near 50 Hz AC equipment on the enzyme’s physicochemical properties has been studied. It was observed that the incubation of the enzyme above the coil of a loaded autotransformer connected to a laboratory benchtop centrifuge led to the enhancement of HRP adsorption onto mica; this enhancement was accompanied by enzyme disaggregation and a slight increase in its activity. Furthermore, incubation near the transformer, which was switched off after its operation and disconnected from the mains power supply, was found to cause even more significant enzyme disaggregation, while the increase in activity was almost the same as in the case with the loaded transformer. Since 50 Hz AC-energized equipment is ubiquitously used in both research and industry, the results obtained in our experiments are quite important for the correct design of experimental procedures and industrial processes involving enzymes.

## 2. Materials and Methods

### 2.1. Chemicals and Enzyme

In our experiments, we used peroxidase from horseradish, which was purchased in the form of a commercial preparation from Sigma (St. Louis, MO, USA; Cat. #P6782; peroxidase from horseradish Type IV-A, essentially salt-free, lyophilized powder; batch No. SLCK8071; enzymatic activity against ABTS 1995 U/(mg solid), RZ 3.0 [48]). In addition, the enzyme was characterized by SDS-PAGE according to the technique reported by Ronzhina et al. [49] as described in the Supplementary Material. According to our data, the major fraction of the enzyme preparation had a molecular weight of 41 kDa (see Figure S1).

The 2,2′-azino-bis(3-ethylbenzothiazoline-6-sulfonate) (ABTS; HRP substrate) was purchased from Sigma (St. Louis, MO, USA; Cat. #A1888). Disodium hydrogen orthophosphate, citric acid and hydrogen peroxide (H_2_O_2_) were purchased from Reakhim (Moscow, Russia). Dulbecco’s modified phosphate-buffered saline (PBSD) was prepared by dissolving a salt mixture, commercially available from Pierce (Appleton, WI, USA), in ultrapure water. All the solutions used in our experiments were prepared using ultrapure deionized water purified with a Simplicity UV system (Millipore, Molsheim, France).

### 2.2. Experimental Setup and Enzyme Treatment

**Figure 1:**
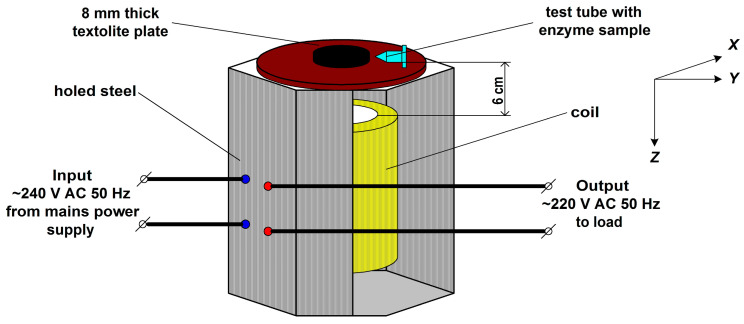
Schematic drawing of the experimental setup. The distance between the transformer’s coil and the test tube is 6 cm.

In order to find out how the incubation near the AC-based equipment affected the HRP enzyme, the setup shown in Figure 1 was employed. The setup included a standard LATR-1 laboratory regulating autotransformer (Russia) and an Eppendorf 5810 R laboratory centrifuge (Eppendorf, Hamburg, Germany). This transformer was based on a toroidal magnetic circuit, which represented a ring-shaped electrical steel core with copper wire winding. The transformer was air-cooled, and rated for a nominal current of up to 9 A.

At the first step of the experiment, the autotransformer was loaded in the following way. The transformer’s input was connected to a 240 V, 50 Hz mains power supply. The output voltage was set to 220 V (Figure 1), and the centrifuge was connected to the transformer output. Then, the centrifuge was switched on and operated at 3000 rpm. The centrifuge power consumption was 1650 W. The centrifuge was located at a distance of 3 m from the transformer. The centrifuge had a metal body with plastic exterior and was grounded. We conducted experiments on the effect of the electromagnetic field of the centrifuge on the aggregation state of the enzyme and did not reveal its effect at a distance of 3 m from the centrifuge and 3 m from the transformer.

After switching the centrifuge on, a test tube with 1 mL of 0.1 µM HRP solution in 2 mM PBSD (the working sample) was placed onto a 8 mm thick textolite plate above the top of the transformer’s coil at a distance of 0.06 m, as shown in Figure 1. At the same time, the control sample was kept at a much larger (10 m) distance from the experimental setup.

The incubation time of the working sample above the loaded transformer upon the centrifuge operation was 30 min. In addition, one enzyme sample was placed into a grounded metal box (a Faraday cage) and incubated therein near the loaded transformer for the same period of time.

At the second step of our experiment, after 30 min of centrifuge operation, the centrifuge was stopped and switched off. The transformer was disconnected from the mains power supply. Five minutes later, another (untreated) working enzyme sample was placed in the same position above the transformer’s coil and incubated there for 30 min. At the points of incubation of the working and control enzyme samples under the conditions of our experiments, no temperature fluctuations exceeding 0.5 °C were registered, as measured with an FY-10 thermocouple-based digital thermometer.

After the above-described procedures, all the enzyme samples studied were subjected to AFM analysis (in order to study the enzyme’s adsorption properties) and to spectrophotometric analysis (in order to determine the enzymatic activity). From each test tube with the studied enzyme samples, 200 µL of enzyme solution was taken for the analysis by spectrophotometry. The remaining 800 µL of each sample solution was used in the AFM analysis procedure described below.

The AFM and spectrophotometry analyses of the enzyme samples were performed in parallel as described in our previous papers—for instance, in [27,28,29]. The procedures, performed throughout these analyses, are briefly described in subsequent sections.

### 2.3. Atomic Force Microscopy Measurements

AFM analysis was performed by the direct surface adsorption method [47]. The adsorption was performed in Eppendorf-type test tubes. Each separate test tube contained an 800 µL volume of either of the samples studied, which were treated as described above in Section 2.2. One AFM substrate (7 mm × 15 mm sheet of freshly cleaved mica; SPI, Charlotte, NC, USA) was immersed into the analyzed enzyme sample solution in either of the test tubes, and incubated there for ten minutes. Each sample was analyzed in three technical replicates. The mica substrates were then scanned in semi-contact mode in air with a Titanium atomic force microscope (NT-MDT, Zelenograd, Russia; the microscope pertains to the equipment of the “Human Proteome” Core Facility of the Institute of Biomedical Chemistry, supported by the Ministry of Education and Science of Russian Federation, Agreement 14.621.21.0017, unique project ID: RFMEFI62117 × 0017). The microscope was equipped with NSG10 cantilevers (TipsNano, Zelenograd, Russia). For each substrate, at least sixteen scans (2 µm × 2 µm in size) were obtained in different areas of the substrate. Then, objects visualized in the so-obtained AFM images were counted with a specialized software custom-developed in IBMC. Based on the number of objects calculated on each AFM substrate (that is, for each enzyme sample studied), distributions of the relative number of objects with height *ρ*(*h*) (density functions) were obtained, and histograms of the absolute number of AFM-visualized particles *N*_400_ (normalized per 400 μm^2^) were plotted vs. the height of the AFM-visualized objects [50]. The standard deviation in the AFM measurements was ≤10%, as obtained for three independent replicates measured for each sample.

### 2.4. Spectrophotometry Measurements and Data Processing

Spectrophotometric analysis was performed based on the well-established technique developed by Sanders et al. [51]. The absorbance of the solution, which contained 1 nM HRP, 0.3 mM azino-bis(3-ethylbenzothiazoline-6-sulfonate) (ABTS) and 2.5 mM H_2_O_2_ in phosphate–citrate buffer (pH 5.0) [51,52], was monitored at 405 nm for 300 s in a 1 cm long quartz cell with an Agilent 8453 spectrophotometer (Agilent Deutschland GmbH, Waldbronn, Gremany) [27,28,29]. For each sample, at least three independent measurements were performed. The results obtained were processed using the least square method as described in [53], and presented in the form of absorbance vs. time (*A_405_*(*t*)) kinetic curves. The standard deviation was calculated using the “*n* − 1” method [53]. The change in HRP enzymatic activity in the working samples relative to the control sample was calculated as

(1) $$∆A\left(working\right)=\frac{A\left(working\right)-A\left(control\right)}{A\left(control\right)}\times 100\%$$

Using Equation (1), the enzymatic activities of HRP in the working and the control enzyme samples were calculated according to the protocol provided by Sigma [54].

### 2.5. Electromagnetic Field Measurements

Both the magnetic and the electric components of the electromagnetic field were measured in the points of location of the working and the control enzyme samples under experimental conditions. The magnetic induction and the electromagnetic field strength were measured with a MEGEON 07020 electromagnetic radiation detector (MEGEON JSC, Moscow, Russia). This detector was not calibrated for measurements of weak alternating electromagnetic fields. A static magnetic field was not detected. The measurements were performed upon positioning the detector’s antenna along three different axes (*X*, *Y*, *Z*) as shown in Figure 1. In each location, five independent measurements were performed.

## 3. Results

### 3.1. AFM Analysis of HRP Adsorption and Aggregation Behaviour

In order to reveal possible changes in the adsorption properties of HRP under experimental conditions, when the enzyme solution was located either near the loaded transformer or near the transformer which was switched off and unplugged from the mains power supply after operation, control experiments were simultaneously carried out to study the aggregation state of HRP. For this purpose, the HRP enzyme solution was placed in test tubes, according to the Materials and Methods Section, away from the experimental setup at a distance of 10 m (control experiments), near the working transformer, and near the transformer after it was turned off (working experiments).

**Figure 2:**
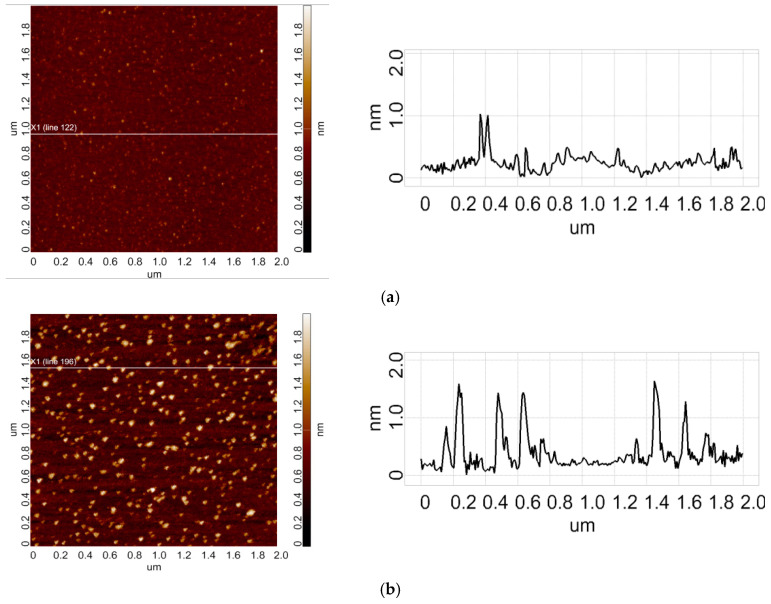

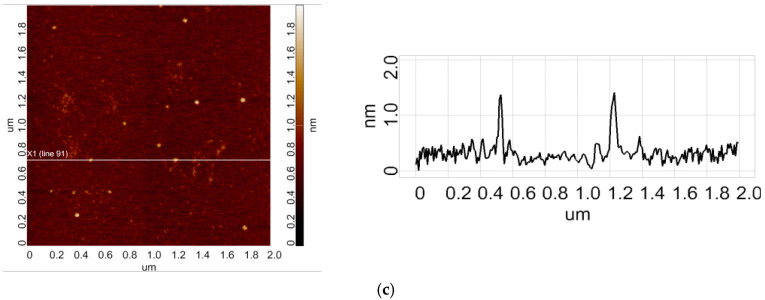
Typical AFM images (**left**) and cross-section profiles (**right**) of HRP adsorbed on mica from the control enzyme sample (**a**), and from working samples incubated near either the loaded (**b**) or the switched-off unplugged autotransformer (**c**) for 30 min.

Figure 2 displays typical AFM images of HRP adsorbed on mica substrate after its incubation in either the control sample (Figure 2a) or the working samples (Figure 2b,c).

The AFM images shown in Figure 2a–c indicate that under our experimental conditions, HRP adsorbs onto mica in the form of compact objects. The AFM images just illustrate the shape of the HRP particles adsorbed on mica. Further analysis was performed in order to exactly determine the effect of incubation of the enzyme near 50 Hz AC equipment on its adsorption behaviour.

**Figure 3:**
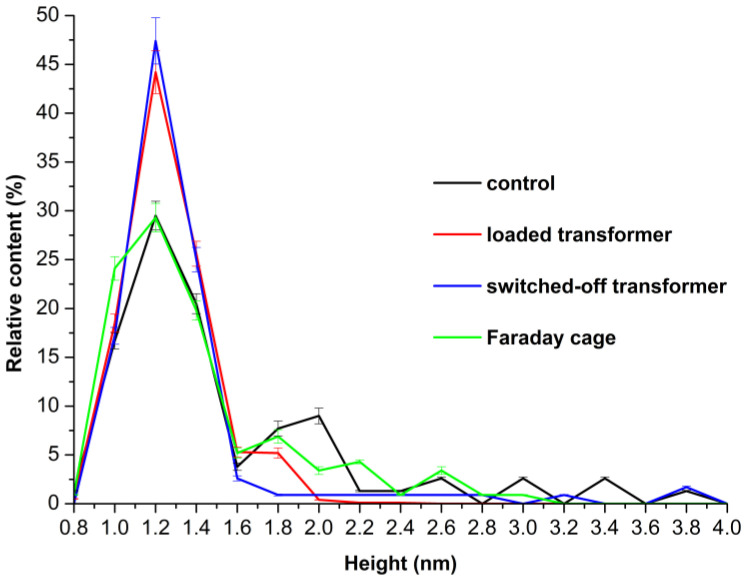
Density function plots *ρ*(*h*) obtained for the enzyme samples studied: the control enzyme sample incubated 10 m away from the experimental setup (black curve); the working sample incubated 0.06 m above the loaded transformer (red curve); the working sample incubated 0.06 m above the switched-off unplugged autotransformer (blue curve); and the sample incubated near the loaded transformer in a Faraday cage (green curve). The incubation time was 30 min.

Figure 3 shows the *ρ*(*h*) plots obtained for the enzyme samples studied.

On the *ρ*(*h*) curve obtained for the control enzyme sample (Figure 3, black curve), two maxima at *h_max_*_1_ = 1.2 ± 0.2 nm and at *h_max_*_2_ = 2.0 nm ± 0.2 nm can be clearly distinguished. Given that the molecular weight of HRP is between 40 and 44 kDa ([55] and our data shown in Supplementary Material, Figure S1) and based on our previously reported observations [27,28], *h_max_*_1_ = 1.2 nm corresponds to the monomeric state of the enzyme, while *h_max_*_2_ = 2 nm corresponds to its oligomeric state. Importantly, the *ρ*(*h*) curve obtained for the sample incubated near the loaded transformer in the Faraday cage has a similar shape, with the first maximum at *h_max_*_1_ = 1.2 ± 0.2 nm, while its second maximum was at *h_max_*_2_ = 1.8 ± 0.2 nm (Figure 3, green curve).

The analysis of *ρ*(*h*) plots obtained for the working sample of the enzyme, which was incubated near the loaded autotransformer, also exhibits a bimodal character with two maxima observed at *h_max_*_1_ = 1.2 ± 0.2 nm and *h_max_*_2_ = 1.8 ± 0.2 nm (Figure 3, red curve). At the same time, the ratio of peak intensities *ρ*(*h_max_*_2_)/*ρ*(*h_max_*_1_) decreased approximately twofold—as compared with that obtained for the control sample. Both facts—this decrease in the *ρ*(*h_max_*_2_)/*ρ*(*h_max_*_1_) ratio observed for the working sample, and the shift of *h_max_*_2_ by ~0.3 nm to the left—clearly indicate a disaggregation of the enzyme after its half-hour-long incubation near the loaded 50 Hz AC transformer. As such, the mica-adsorbed HRP represents a mixture of its monomeric and aggregated forms in both cases described.

At the same time, the results obtained upon the analysis of the working sample incubated near the switched-off unplugged transformer were very interesting; namely, the *ρ*(*h*) plot obtained for this sample was quite different from the ones discussed above. It demonstrated a unimodal distribution function with a maximum at a height of *h_max_*_1_ = 1.2 ± 0.2 nm, being devoid of the second maximum (Figure 3, blue curve). That is, the half-hour-long incubation of the sample near the unloaded, switched-off 50 Hz AC transformer, which had been disconnected from the mains power supply, led to predominant adsorption of the monomeric form of the enzyme.

**Figure 4:**
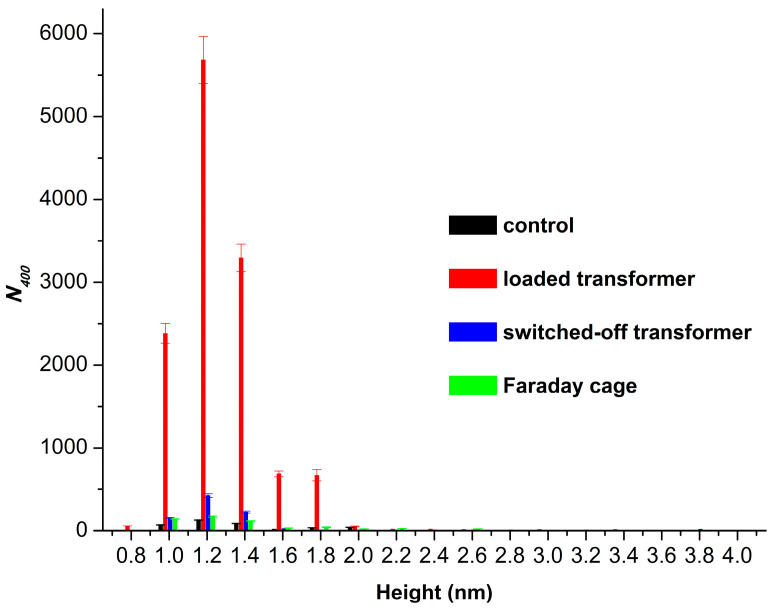
Histograms of absolute number of AFM-visualized particles, normalized per 400 µm^2^, vs. height, obtained for the enzyme samples studied: the control enzyme sample incubated 10 m away from the experimental setup (black bars); the working sample incubated 0.06 m above the loaded transformer (red bars); the working sample incubated 0.06 m above the switched-off unplugged autotransformer (blue bars); and the sample incubated near the loaded transformer in a Faraday cage (green bars). The incubation time was 30 min.

In addition, the absolute number of HRP particles adsorbed on mica in our experiments was considered. Figure 4 displays the histograms of the absolute number of AFM-visualized particles, normalized per 400 µm^2^, vs. height, obtained for the control and the working samples.

The histograms shown in Figure 4 indicate that only 435 particles per 400 µm^2^ were adsorbed from the control enzyme sample. In the case with the sample incubated near the loaded transformer in the Faraday cage, the number of adsorbed particles was similar and amounted to 575 particles per 400 µm^2^. At the same time, from the working sample incubated near the loaded transformer, the number of adsorbed particles increased by two orders of magnitude to 12,858 objects per 400 μm^2^. And when the enzyme solution was incubated near the switched-off, unplugged transformer, the number of visualized objects was similar to that observed for the control sample, amounting to 895 particles per 400 µm^2^.

Summarizing the results of our AFM experiments, we can conclude that the half-hour-long incubation of the HRP solution near a loaded 50 Hz AC transformer promoted a disaggregation of the enzyme, accompanied by a great enhancement in its adsorption. However, the incubation near the switched-off unplugged transformer resulted in a more considerable disaggregation of the enzyme, while its adsorption increased much less significantly.

### 3.2. Spectrophotometric Estimation of Enzymatic Activity

**Figure 5:**
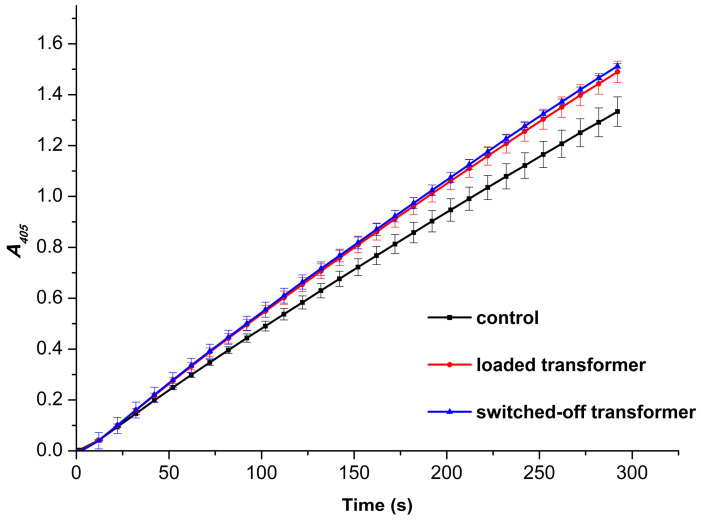
*A_405_*(*t*) kinetic curves obtained for the HRP-ABTS-H_2_O_2_ system. The curves were recorded for the control enzyme sample (black curve), and for the working samples incubated above either the loaded (red curve) or switched-off unplugged transformer for half an hour.

In parallel, the influence of incubation of HRP near 50 Hz equipment on its enzymatic activity against ABTS was determined by spectrophotometry, as is briefly described in the Materials and Methods. Figure 5 displays typical *A_405_*(*t*) kinetic curves obtained for the control and the working samples studied by AFM.

The *A*_405_(*t*) kinetic curves shown in Figure 5 clearly indicate an increase in the enzymatic activity of HRP against its substrate ABTS after the incubation of the enzyme solution above the transformer. It is quite interesting to note that there is no difference in the activity between the two working samples. That is, the incubation of the enzyme near either the loaded or the switched-off transformer led to virtually the same increase in its activity. Namely, the enzymatic activity of HRP in the control sample amounted to 74.48 ± 3.24 units/(mL enzyme), while the activity of the enzyme increased to 83.20 ± 2.35 units/(mL enzyme) and 84.48 ± 0.56 units/(mL enzyme) after the half-hour-long incubation near the loaded transformer and near the switched-off transformer, respectively. In other words, the incubation of the enzyme near the loaded or near the switched-off unplugged transformer resulted in 12% and 13% increases in its activity, respectively. In addition, the standard deviation did not exceed 4.35%.

### 3.3. Results of Electromagnetic Field Measurements

Both the magnetic and the electric components of the electromagnetic field were measured in the points of location of the working and the control enzyme samples under experimental conditions. Our measurements indicated the absence of electromagnetic radiation within the frequency range from 5 Hz to 3.5 GHz in both the point of the control sample incubation and above the unplugged transformer. Similarly, both the magnetic induction and the electric field strength were equal to zero within the Faraday cage. In contrast, considerable electromagnetic radiation was detected at the point of incubation of the enzyme sample above the transformer’s coil when the transformer was loaded as was described in the Materials and Methods. The respective data are listed in Table 1.

## 4. Discussion

In this work, we have investigated how incubation near 50 Hz AC-energized equipment influences the physicochemical properties of the HRP enzyme. In our experiments, we revealed that the half-hour-long incubation of 0.1 µM HRP solution at a 6 cm distance from the loaded transformer led to a disaggregation of the enzyme. This disaggregation was accompanied by both an enhancement in the number of adsorbed enzyme particles and a 12% increase in its activity against ABTS substrate. Furthermore, we observed a nearly complete disaggregation of the adsorbed enzyme (Figure 3, blue curve vs. black curve) accompanied by a similar (13%) increase in its activity (Figure 5, blue curve vs. black curve) after the incubation of the enzyme solution near the switched-off unplugged transformer. One of the factors which might influence the enzyme incubating near the experimental setup is sound. The centrifuge operation is accompanied by sound, and this factor may explain the difference between the *ρ*(*h*) curves obtained for the control enzyme sample (Figure 3, black curve) and the sample incubated in the Faraday cage (Figure 3, green curve). The possible influence of sound waves on the enzyme requires additional investigation. Another factor is the influence of electromagnetic fields: HRP is known to be sensitive to the action of electromagnetic and magnetic fields [2,3,4,5,6,7,18,21,27,28,29]. Though the latter phenomenon might be explained by the influence of residual magnetism (also known as remanence) in the autotransformer’s core on the enzyme, the additional checks of both the electric and the magnetic field indicate the absence of remanence-induced fields in the point where the enzyme solution was incubated above the transformer’s coil, as shown in Figure 1. A static magnetic field has not been registered. Despite residual magnetism may sometimes remain in the transformer’s core for quite a long time [56], which can reach several hours [57], this was not the case with respect to our situation. HRP is sensitive to the action of magnetic fields [2,6,7,18], which can result in its activation [18].

In this connection, we should emphasize that the exposure of HRP to electromagnetic or magnetic fields of either low (typically below 100 Hz [2,4,5]) or radio [21] frequencies often causes a change in its enzymatic activity. The effect of these fields on the enzyme depends on the experimental conditions—namely, on the enzyme solution acidity [2], the exposure time [2,5,21], and the field parameters. The combination of these factors determines whether the enzymatic activity will be enhanced or suppressed by the field. In our case, the half-hour-long exposure of 0.1 µM HRP solution in Dulbecco’s modified phosphate-buffered saline (pH 7.4) resulted in a 12–13% increase in the enzymatic activity against ABTS. A similar (by 5.33 to 13.73%) enhancement of HRP activity against guaiacol was reported by Yao et al. [21] after a 2.5 to 4.5 min long radio frequency heating of 0.1 mg/mL HRP solution to 50 °C. These authors observed a deactivation of the enzyme upon heating to higher (70 °C and 90 °C) temperatures, which was achieved by increasing the heating time [21]. Wasak et al. [2] reported that the treatment of HRP in a rotating magnetic field at a pH of 4.5 enhances its enzymatic activity at 1 Hz and 20 Hz field frequencies, while at the other frequencies studied (2 to 50 Hz, except 20 Hz), the enzymatic activity of HRP was suppressed. Emamdadi et al. [18] emphasized the considerable activation of HRP by a static magnetic field. Caliga et al. [4] and Portaccio et al. [5] also reported a suppressing effect of a 50 Hz electromagnetic field on HRP activity.

The changes in adsorption, aggregation and catalytic properties of HRP under the influence of a 50 Hz electromagnetic field can be explained by a re-distribution of the hydration shells of enzyme particles. Indeed, changes in enzyme hydration are known to be the causes of alterations in enzymatic activity [58,59,60]. Furthermore, it is no surprise that the disaggregation of the enzyme is accompanied by an increase in its catalytic activity: enzyme aggregation is commonly ascribed to a loss of activity [61,62,63], and we have observed the reverse process. The changes in the enzyme hydration also influence both enzyme–enzyme [64] and enzyme–AFM substrate surface [65] interactions by changing the balance between electrostatic, van der Waals and hydration repulsion interactions [66,67,68,69,70], thus affecting the enzyme adsorption on mica. This is how we explain the phenomena observed in our experiments.

Once again, in this work, we have discovered that the properties of the enzyme are affected not only by the electromagnetic fields emitted by energized equipment (with the example of a loaded transformer), but even after the exposure of the enzyme to switched-off 50 Hz AC equipment. In the situation with the loaded autotransformer, the increase in enzyme adsorption onto the mica surface could be explained by the effect of the 50 Hz electromagnetic field, which could cause a change in the enzyme hydration, leading to an increase in the adsorption properties of the enzyme to the mica surface. Indeed, both the electric field strength and the magnetic induction near the loaded transformer were considerable (see Table 1), and the HRP enzyme is sensitive to the influence of electromagnetic fields [2,3,4,5,6,7,18,21,27,28,29], explaining this quite significant change in its adsorption (Figure 4, red bars). But when the autotransformer was switched off and disconnected from the mains power supply, incubation above the transformer’s coil resulted in an even more significant decrease in the degree of enzyme aggregation. We should emphasize that both the transformer and the centrifuge were shut down in this case, so no sound was generated by the experimental setup. Accordingly, neither the influence of residual magnetism nor the sound impact can explain this phenomenon, which thus requires future thorough investigation. Bunkin et al. [71] ascribed the post-effect of low-frequency radiation in the aqueous medium to the formation of nanobubble clusters. This process was observed during the electromagnetic excitation of the aqueous medium by an external electromagnetic field. Such a bubble formation was confirmed in other papers [72,73,74].

## 5. Conclusions

Herein, by using atomic force microscopy (AFM) and spectrophotometry analysis performed in parallel, we have investigated how incubation near 50 Hz AC equipment influences the physicochemical properties of horseradish peroxidase (HRP). We have found that half-hour-long incubation of the enzyme 6 cm above the coil of a loaded autotransformer promotes a disaggregation of HRP on mica with a simultaneous enhancement of the number of mica-adsorbed enzyme particles by two orders of magnitude as compared with the control sample. Density function plots obtained for both the control and the working sample of the enzyme incubated near the loaded autotransformer exhibited a bimodal character with two maxima. For the control sample, the maxima were observed at *h_max_*_1_ = 1.2 ± 0.2 nm and at *h_max_*_2_ = 2.0 nm ± 0.2 nm, while for the working sample the maxima were observed at *h_max_*_1_ = 1.2 ± 0.2 nm and *h_max_*_2_ = 1.8 ± 0.2 nm. At the same time, the ratio of peak intensities *ρ*(*h_max_*_2_)*/ρ*(*h_max_*_1_) decreased approximately twofold as compared with that obtained for the control sample. Both the decrease in the *ρ*(*h_max_*_2_)*/ρ*(*h_max_*_1_) ratio, and the shift of *h_max_*_2_ by ~0.3 nm to the left observed for the working sample, clearly indicate a disaggregation of the enzyme after its half-hour-long incubation near the loaded 50 Hz AC autotransformer.

Most interestingly, the incubation of HRP above the switched-off unplugged transformer for the same period of time was found to cause an even more significant disaggregation of the enzyme! Namely, the density function plot obtained for this sample exhibited a unimodal character with a maximum at a height of *h_max_*_1_ = 1.2 ± 0.2 nm, being devoid of the second maximum. The effect on the amount of mica-adsorbed enzyme particles was much less significant in the latter case. But at the same time, a 12 to 13% increase in the enzymatic activity of HRP was observed in both cases. Thus, the half-hour-long incubation of the enzyme near the switched-off unplugged autotransformer also had a clearly distinguishable effect on both the adsorption properties and the activity of the enzyme. The latter phenomenon requires further thorough investigation. The effects reported in our manuscript emphasize the importance of consideration of the influence of low-frequency electromagnetic fields on enzymes in the design of laboratory and industrial equipment involving enzyme systems. Namely, if any laboratory or industrial equipment intended for operation with enzymes contains transformers, they must be properly ground-shielded in order to avoid the undesired effects of the transformer-induced electromagnetic fields on the enzymes processed. Our results reported herein can be interesting to scientists studying enzyme systems, and to engineers developing the laboratory and industrial equipment, which is intended for operation with enzymes.

## Figures and Tables

**Table 1:** Results of measurements of the electromagnetic field at the point of incubation of the HRP sample above the loaded autotransformer.

Detector’s Antenna Orientation Axis *	Electric Field Strength (V/m)	Magnetic Induction (µT)
*X*	97.4 ± 14.7	21.4 ± 10.9
*Y*	110.6 ± 3.5	80.6 ± 5.4
*Z*	179.0 ± 11.0	9.3 ± 4.2

* The directions of the *X*, *Y* and *Z* axes are shown in Figure 1.

## Data Availability

Correspondence and requests for materials should be addressed to Y.D.I. The data underlying the research can be provided upon request by the corresponding author.

## Supplementary Materials

The following supporting information can be downloaded at: https://www.mdpi.com/article/10.3390/mi16030344/s1, Figure S1: The image of the gel obtained in the SDS-PAGE analysis of the HRP preparation used in the study; Table S1: Preparation of polyacrylamide gels.
