# Supplementary material for: Incubation of Horseradish Peroxidase near 50 Hz AC Equipment Promotes Its Disaggregation and Enzymatic Activity
Source: Micromachines (Basel). 2025 Mar 19;16(3):344. doi: 10.3390/mi16030344 (PMC11944298; doi:10.3390/mi16030344)
Supplement: Supplementary file 1 [file micromachines-16-00344-s001.zip › micromachines-3383044-supplementary.pdf]

Supplementary Information to:

Incubation of horseradish peroxidase near 50 Hz AC equipment promotes its disaggregation and enzymatic activity

by

Yuri D. Ivanov [1,2]*, Ivan D. Shumov [1], Andrey F. Kozlov[1], Alexander N. Ableev[1], Angelina V. Vinogradova[1], Ekaterina D. Nevedrova[1], Oleg N. Afonin[1], Dmitry D. Zhdanov[1], Vadim Y. Tatur[3], Andrei A. Lukyanitsa[3, 4], Nina D. Ivanova[3,5], Evgeniy S. Yushkov[6], Dmitry V. Enikeev[7], Vladimir A. Konev[8] and Vadim S. Ziborov[1,2]

1 Institute of Biomedical Chemistry, Pogodinskaya Str., 10 Build. 8, 119121 Moscow, Russia; shum230988@mail.ru (I.D.S.); afkozlow@mail.ru (A.F.K.); ableev@mail.ru (A.N.A.); angeluna1234@bk.ru (A.V.V.); nevedrova.kat@yandex.ru (E.D.N.); sunweb@mail.ru (O.N.A.); zhdanovdd@gmail.com (D.D.Z.); ziborov.vs@yandex.ru (V.S.Z.)

2 Joint Institute for High Temperatures of the Russian Academy of Sciences, 125412 Moscow, Russia

3 Foundation of Perspective Technologies and Novations, 115682 Moscow, Russia; v_tatur@mail.ru (V.Y.T.); andrei_luk@mail.ru (A.A.L.); ninaivan1972@gmail.com (N.D.I.)

4 Faculty of Computational Mathematics and Cybernetics, Moscow State University, 119991 Moscow, Russia

5 Moscow State Academy of Veterinary Medicine and Biotechnology Named after Skryabin, 109472 Moscow, Russia;

6 Department for Business Project Management, National Research Nuclear University "MEPhI", 115409 Moscow, Russia; esyushkov@mephi.ru

7 Institute for Urology and Reproductive Health, I.M. Sechenov First Moscow State Medical University (Sechenov University), 119991 Moscow, Russia; dvenikeev@gmail.com

8 Department of Infectious Diseases in Children, Faculty of Pediatrics, N.I. Pirogov Russian National Research Medical University, 117997 Moscow, Russia; konev60@mail.ru

* Correspondence: yurii.ivanov.nata@gmail.com

Electrophoresis in polyacrylamide gel under denaturing conditions (SDS-PAGE) procedure

To prepare the gels, a casting chamber with 80×90×1 mm dimensions (Gel caster, Amersham Biosciences, USA) was used. Tris-HCl solution, used for preparation of the gel, was prepared preliminarily, filtered through a 0.45 μm filter (Schleicher & Schuell) and stored at 4°C.

For separation by molecular weight, polyacrylamide gel plates (13.5%) prepared as shown in Table S1, were used.

Table S1. Preparation of polyacrylamide gels.

	**Concentrating gel**	**Separating gel**
Concentration [%]	5	13.5
40% acrylamide [mL]	0.6	10.1
1M Tris-HCl (pH 6.8) [mL]	1.25	-
1.5M Tris-HCl (pH 8.8) [mL]	-	7.5
10% ammonium persulfate) [mL]	0.05	0.3
Temed [mL]	0.005	0.015
H_2O [mL]	3.1	12.2
Final volume [mL]	10	30

Firstly, the chamber was filled with the separating gel solution. The separating gel was allowed to polymerize for 30-50 min, and then the glasses with the gel were taken out, washed with water, combs were inserted and wells were formed by pouring in the solution for the concentrating gel. Combs with a tooth width of 5 mm were used to form pockets for the samples. The concentrating gel was allowed to polymerize for 30-40 min.

The analyzed samples were added to sample buffer (2% LDS, 0.065M Tris, pH 6.8, 1% DTT, 10% glycerol, 0.01% bromophenol blue) and heated in a boiling water bath for 2 min. A preliminarily prepared sample buffer was used at fivefold concentration and stored in aliquots at (-20)˚C.

Solutions of molecular weight markers and the analyzed enzyme sample were dispensed into the wells of the gel as follows (see Figure S1): 5 μL of Precision Plus Protein Standards molecular weight marker (Bio-Rad, USA); 24 μL of the analyzed HRP enzyme sample (0.09 mg/mL) + 6 μL of 5xLDS; 1 mg/mL BSA.

Electrophoresis was performed with a Hoefer miniVE system (Amersham Biosciences, USA) at room temperature. The electrophoresis parameters were as follows: 85 V for 20 min (1); 100 V for 20 min (2); 130 V for 2 h 16 min, i.e. until the front reaches the lower edge of the gel.

Upon completion of the electrophoresis, the polyacrylamide gel was placed into a fixing solution (25% isopropanol, 10% acetic acid) for 15 min twice. The gel was stained for one hour with 0.1% Coomassie R250 solution in 30% methanol containing 10% acetic acid. Upon completion of staining, the background was decolorized by repeated wash with water (with change every 30 min until a satisfactory result was obtained).

The so-obtained gel was scanned with an ImageScaner III (GE Healthcare, USA) scanner, and the resulting image is shown in Figure S1.

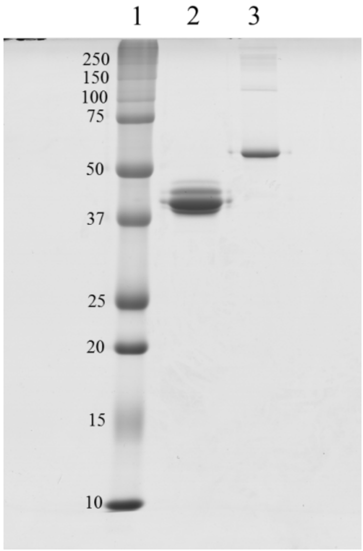

Figure S1. The image of the gel obtained in the SDS-PAGE analysis of the HRP preparation used in the study. Molecular weights of the molecular weight markers are indicated at the left. Solutions of the molecular weight markers and the analyzed enzyme sample were dispensed into the wells of the gel as follows: 5 μL of Precision Plus Protein Standards molecular weight marker (Bio-Rad, USA) (Lane 1); 24 μL of the analyzed HRP enzyme sample (0.09 mg/mL) + 6 μL of 5xLDS (Lane 2); 1 mg/mL BSA (Lane 3).

The analysis of the gel image indicated that during the electrophoretic separation, four protein bands of the sample under study are visualized. The molecular weights of these fractions were 39, 41, 44 and 47 kDa, respectively. The highest value of molecular weight was 47 kDa. One can clearly see that the third band had the highest intensity. Accordingly, one can conclude that the molecular weight of the major fraction of the HRP enzyme preparation studied corresponded to 41 kDa.
